# Differential Expression of Anti-Inflammatory RNA Binding Proteins in Lupus Nephritis

**DOI:** 10.3390/life12101474

**Published:** 2022-09-23

**Authors:** Raouia Fakhfakh, Emna Bouallegui, Hana Houssaini, Nesrine Elloumi, Fatma Dhafouli, Olfa Abida, Hend Hachicha, Sameh Marzouk, Zouhir Bahloul, Khawla Kammoun, Tahia Boudawara, Hatem Masmoudi

**Affiliations:** 1 Autoimmunity, Cancer, and Immunogenetics Research Laboratory, LR18SP12, University Hospital Habib Bourguiba of Sfax, Sfax 3000, Tunisia; 2 Department of Immunology, Habib Bourguiba University Hospital, University of Sfax, Sfax 3000, Tunisia; 3 Internal Medicine Department, Hedi Chaker University Hospital, University of Sfax, Sfax 3000, Tunisia; 4 Renal Pathology Research Unit 12ES14, Nephrology Department, Faculty of Medicine of Sfax, Hedi Chaker University Hospital, Sfax 3000, Tunisia; 5 Department of Pathology, LR18SP10, Habib Bourguiba University Hospital, Sfax 3000, Tunisia

**Keywords:** lupus nephritis, RNA binding protein, cytokines

## Abstract

Lupus nephritis (LN) is a type of immunological complex glomerulonephritis characterized by chronic renal inflammation which is exacerbated by infiltrating leukocytes and fueled by a variety of pro-inflammatory cytokines. A profound understanding of the pathogenesis of LN is necessary to identify the optimal molecular targets. The role of RNA-binding proteins (RBPs) in post-transcriptional gene regulation in the immune system is being explored in greater depth to better understand how this regulation is implicated in inflammatory and autoimmune diseases. Tristetraprolin (TTP), Roquin-1/2, and Regnase-1 are 3 RBPs that play a critical role in the regulation of pro-inflammatory mediators by gating the degradation and/or translational silencing of target mRNAs. In this study, we proposed to focus on the differential expression of these RBPs in immune cells and renal biopsies from LN patients, as well as their regulatory impact on a specific target. Herein, we highlight a novel target of anti-inflammatory treatment by revealing the mechanisms underlying RBP expression and the interaction between RBPs and their target RNAs.

## 1. Introduction

Lupus nephritis (LN) is an organ manifestation of systemic lupus erythematosus (SLE), a prototypic inflammatory autoimmune disorder. LN is developed in more than 50 % of SLE patients. It is the strongest predictor of morbidity and mortality, responsible for the greater burden attributable to the SLE disease, mainly in low-income countries, such as Tunisia. LN is characterized by extensive immune inflammation, and fluctuating disease activity [1]. It is distinguished by the presence of multiple auto-antibodies (Abs), immune complex (IC) deposition, and complement system activation, which results in kidney damage [2]. Peripheral blood mononuclear cells (PBMCs), containing mainly T and B cells, are prominent sentinels of LN immune responses. Recently, several studies demonstrated that polymorphonuclear neutrophils (PMNs), the most abundant type of circulating leukocytes and the first innate immune effector cell to be recruited to sites of infection, contribute to active LN by circulating neutrophil-derived inflammatory peptides and cytokines [3].

Therefore, a key step in establishing LN as well as in preventing tissue damage is the control of the expression of inflammatory mRNAs in immune cells, particularly post-transcriptional regulation [4,5]. This regulation, including RNA editing, localization, stability, and translation, is coordinated by a range of RNA-binding proteins (RBPs) [6]. Among them, a class of RBPs has been demonstrated to be involved in the regulation of inflammation and immune response, and autoimmune pathogenesis, especially in mouse models, including Regnase-1 (also known as MCPIP-1, encoded by *ZC3H12A* gene) [7], TTP (Tristetraprolin, encoded by *ZFP36* gene) [8] and the Roquins (Roquin-1 and 2, encoded by *RC3H1* and *RC3H2* genes respectively) [9]. Regnase-1 regulates mRNA expression upon TLR induction and participates in the control of the acute phases of inflammation by promoting the decay of a set of proinflammatory cytokines mRNAs, such as IL6 [10,11]. In CD4^+^ T cells, loss of Regnase-1 leads to markedly elevated IL-6 levels and, consequently, exacerbated autoimmune disease risk as demonstrated in the experimental autoimmune encephalitis model of multiple sclerosis [12]. Recently and through its pro-apoptotic activity, Regnase-1 has been implicated in the induction of neutrophil apoptosis [13]. Moreover, TTP prevents the establishment of severe inflammation and is involved in anti-inflammatory responses by inhibiting the production of tumor necrosis factor (TNF)-α [14]. It is also involved in the control of neutrophil accumulation and survival [15]. Likewise, the Roquin family are rather new members of the RBPs. This family is formed by Roquin-1 and its paralogue, Roquin-2. These two proteins contribute to the maintenance of immune tolerance and the prevention of inflammation and autoimmune diseases by post-transcriptional regulation of the expression of different cytokines mRNAs such as *TNF-α* and *IL6*, mRNAs of costimulatory molecules including Inducible T Cell Costimulator (ICOS) and *OX40*, as well as mRNAs of transcription factors [16]. However, these RBPs have not yet been shown to be related to susceptibility to autoimmune diseases, such as kidney disease, in humans, with a few exceptions [17,18]. 

Our research aims to better understand the role of RBPs in the pathophysiology of LN. First, we examined the expression of their respective genes and their targets, *IL-6*, *TNF-α*, and *ICOS*, in blood cells with or without pathogen components stimulation in LN patients compared with healthy controls (HC), and second, we studied the RBPs tissue expression in renal biopsies.

## 2. Material and Methods

### 2.1. Patients and Controls

Twenty-seven LN patients have been recruited since 2017 from the Internal Medicine and Nephrology Departments of the Hedi Chaker University Hospital of Sfax, Tunisia. All patients fulfilled the revised criteria of the American College of Rheumatology for SLE [19]. Renal disease was defined as proteinuria >0.5 g/24 h and/or urine sediment abnormalities, such as glomerular haematuria or aseptic leukocyturia and/or renal failure. LN was diagnosed by renal biopsy and classified according to the International Society of Nephrology/Renal Pathology Society classification of LN (ISN/RPS) 2003 revised classification system [20]. The disease activity was assessed in LN patients by the Systemic Lupus Erythematosus Disease Activity Index (SLEDAI) [21]. For all patients, the results of immunological tests (anti-nuclear antibodies (ANA), anti-DNA Abs) were collected and performed for daily routine. The 27 patients included in our study are SLE patients who present as newly diagnosed LN patients in the active phase of their disease. All treated patients were under a weak dose of corticoid treatment (not over 5 mg/day) and no one of them was receiving immunosuppressive drugs for over 3 months. LN Patients with SLE-related and other nonspecific illnesses (Sy Sjögren’s syndrome or discoid lupus erythematosus), as well as patients with poor medical data or who did not get regular follow-ups, were excluded.

This study was approved by the ethical committee of the Habib Bourguiba Hospital of Sfax, Tunisia (protocol number of the ethical committee, 02/14). After obtaining written informed consent from the participants before enrolment into the study, samples were collected:

(i) Blood: A total of 10 mL of blood was taken from LN patients (n = 9) and HC (n = 9) in sterile endotoxin-free vacutainers with EDTA as an anticoagulant for mRNA expression analysis.
(ii) Biopsies: 18 paraffin-embedded LN renal biopsies were collected from the department of pathology, Habib Bourguiba University Hospital, Sfax, Tunisia. LN renal biopsies with class VI (where more than 90% of glomeruli present global glomerulosclerosis) and sections containing several glomeruli less than 5 in hematoxylin-eosin (HE) staining were excluded from the study. Clinical, serological, and histological data (using Abs against human IgG, IgM, IgA, C1q, C3, kappa and lambda light chains, albumin and fibrinogen) of biopsies were collected at the same time as the biopsy. The biopsies were divided into 2 groups according to the proliferative status (i) Severe proliferative group (G1) including biopsies with class III (n = 1), class IV (n = 4), class III+V (n = 4), and class IV+V (n = 6) and (ii) non-severe proliferative group (G2) including biopsies with class II+V (n = 1) and V (n = 2). The activity and sclerosis index were used to assess disease activity and cumulative damage.

As controls, 9 paraffin-embedded normal renal tissues, from 5 patients who underwent nephrectomy due to renal carcinoma and from 4 cadaver subjects without any renal disease who underwent autopsy kidneys, were obtained. The absence of cellular infiltrate and inflammation has been confirmed by histological examination by HE staining.

### 2.2. Immune Cells Isolation and Stimulation

On the same day as the blood sampling, PBMCs and PMNs were isolated by removing of red blood cells (RBC) using 2% dextran-promoted rosette formation and hypotonic lysis (0.2% NaCl followed by 1.6% NaCl). PMNs were separated from PBMCs using two discontinuous density gradients of Ficoll-Paque premium. After isolation, the viability and the purity of PBMCs and PMNs were checked and evaluated by trypan and truck blue respectively in light microscopy. Lipopolysaccharide (LPS) (*Escherichia coli* serotype O111:B4, Cell Signaling Technology^®^, Massachusetts, MA, USA) was used to activate MAPK and NFκB-dependent signaling pathways, inducing inflammation-related genes encoding pro- and anti-inflammatory cytokines and therefore, the RBPs expression among cultured blood cells. For this, 2 × 10^6^ cells/mL were placed into dedicated wells of 24-well plates, and PMNs or PBMCs were incubated for 1 or 2 h, respectively, in RPMI medium containing LPS (100 ng/mL) at 37 °C. The stimulation condition was fixed after a preliminary experience in which we used a non-cytotoxic dose of LPS (1–100 ng/mL) for 1 h, 2 h, and 3 h stimulation in PBMCs (data not shown). Cells cultured under similar conditions without stimulation served as controls. The basal condition, unstimulated, and stimulated cell suspension was lysed with Trizol reagent (Invitrogen, Waltham, MA, USA) and stored in Eppendorf tubes at −80 °C.

### 2.3. Total RNA Isolation and Semi-Quantitative RT-PCR for Gene Expression

Total RNA was extracted from the Trizol suspension according to the manufacturer’s instructions. The RNA purity and integrity in each sample were assessed using a NanoDrop system (NanoDrop Technologies^®^, Wilmington, NC, USA) and using standard agarose gel electrophoresis. RNA was reversely transcribed using the PrimeScript RT Reagent Kit (TAKARA Bio Inc.^®^, Shiga, Japan). Gene expression of the 4 RBPs and their targets *TNF-α*, *Il-6*, and *ICOS*, were analyzed, using Gene-specific primers, by SYBR Green Dye detection system analysis in real-time quantitative PCR (RT–PCR) (Table S1). All reactions were performed in duplicate. For verification of the quality of PCR products, melting curves were generated. The relative quantification was performed using the standard curve method and data were analyzed by the comparative 2^−ΔCt^ method and normalized to the average housekeeping gene GAPDH. 

### 2.4. Immunohistochemistry for Protein Expression

All biopsies were taken for Regnase-1, TTP, and Roquins immunohistochemically (IHC) staining as described in Elloumi et al. [22]. In short, after being assembled on slides and deparaffinized, endogenous peroxidase was blocked with 3% hydrogen peroxide for 5 min; Sections were incubated for 16 h with anti-Regnase-1 (MPA032052, Atlas Antibodies^®^, Stockholm, Sweden), anti-TTP (ab124024, Abcam^®^, Cambridge, UK), and anti-RC3H2 (PA5-60151, Invitrogen^®^ Sweden) Abs at 1:200 dilutions and with anti-RC3H1 Ab (ab244405, Abcam) at 1:500 dilutions. After 30 min of incubation with secondary Abs [a soursop peroxidase (HRP)-IgG-anti-rabbit designed by Novolink Polymer (Leica Biosystems^®^, Lincolnshire, IL, USA)], a prepared chromogenic solution of diaminobenzidine (DAB) substrate was incubated for 10 min. Finally, the sections were counterstained lightly with hematoxylin and examined under a light microscope (Axiolab, Zeiss^®^, Toronto, ON, Canada). For negative control preparation, the primary Abs were replaced with irrelevant isotype-matched control immunoglobulin (data not shown). The dilution and the incubation time of each Ab were fixed following marking on a section of the pancreas used as a positive control according to the manufacturer’s recommendations (Figure S1). Photographic images of representative results were captured using a Zeiss^®^Axiocam color camera, and Canon^®^ A 640 Power Shot camera. Semi-quantitative analysis was performed by light microscopy and the interpretation was carried out by a pathologist and a nephrologist.

### 2.5. Biopsy IHC Scoring

We determined three scores for each sample; distribution score, intensity score, and an expression score resulting from the product: intensity score X distribution score based on the strategy adopted in the studies of Elloumi et al. [22,23]. The Intensity Score ranged from 0 to 3: 0 for negative, 1 for weakly positive, 2 for moderately positive, and 3 for strongly positive staining. The Distribution Score ranged from 0 to 4 depending on the stained surface of glomeruli: the score was 0 for 0%, 1 for 0% to 25%, 2 for 25% to 50%, 3 for 50% to 75%, and 4 for more than 75%. 

### 2.6. Statistical Analysis

Data were analyzed using SPSS version 20; the non-parametric Mann–Whitney *U*-test to compare the RBP and their target genes expression between LN patients and controls in immune cells and renal tissues. The logarithms of mean expression levels were used in Mann–Whitney *U*-tests of qPCR data. Mann–Whitney *U*-test was also used to study the association between RBP expression and qualitative clinical, serological, and histological features of LN. While, for the correlation between RBP expression and quantitative features of the disease, we used the Spearman correlation. the Mann–Whitney *U*-test was used to analyze the difference in RBP expression in renal biopsies between HC and LN patients. Statistical differences between the different classes were analyzed using a one-way analysis of variance followed by post hoc Tukey’s multiple comparison tests. For correlation studies, the Spearman rank correlation was used, and for the association studies between the expression score and the absence or presence of Abs deposit, we used the Mann–Whitney U-test. A *p*-value less than 0.05 was considered statistically significant.

## 3. Results

### 3.1. Demographic and Clinical Characteristics of the Studied Patients and Controls

Twenty-seven patients with active LN aged 35 ± 10 years and 9 age-matched HC (age 30 ± 12 years) were enrolled in this study. The majority of patients in this study were females with a ratio F/M of 8:1. The different clinical manifestations and hematological and immunological features are summarized in Table 1. All LN patients, included in our study, were with SLEDAI ≥ 10, reflecting a high disease activity group. Most patients suffered from leucopenia, malar rash, ocular ulcers as well as anemia. The age of LN onset varied between 3 and 10 years (mean 9.25 ± 4.501), at the time of diagnosis. ANA were detected in all patients. Both C3 and C4 levels were low with a mean value at 0.312 g/L (0.05–0.76) and 0.149 g/L (0.05–0.39) respectively. A high level of anti-dsDNA Abs was found at 155.32 ± 186.1.

### 3.2. RBP Transcription Levels in Immune Cells

**Figure 1:**
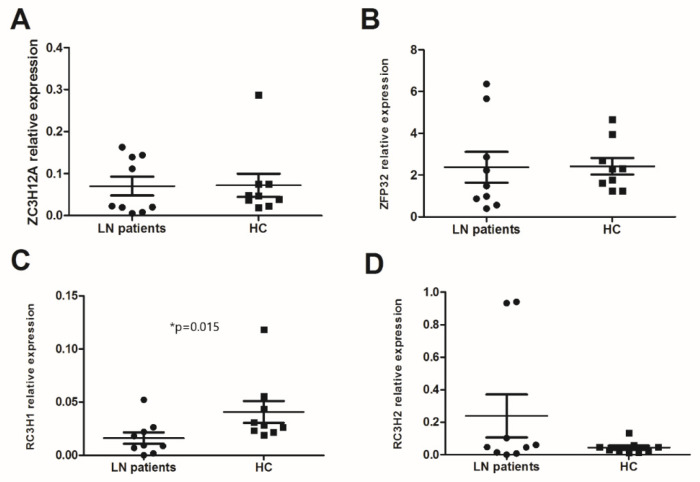
mRNA levels of anti-inflammatory RNA-binding proteins (RBPs) in peripheral blood mononuclear cells (PBMCs): mRNA expression levels of (**A**) *ZC3H12A* (Regnase-1 gene), (**B**) *ZFP36* (TTP gene), (**C**) *RC3H1* (Roquin-1 gene), and (**D**) *RC3H2* (Roquin-2 gene) were determined by semi-quantitative reel time PCR in PBMCs from lupus nephritis (LN) patients (n = 9) and healthy controls (HC) (n = 9) after normalization with GAPDH mRNA level. Data were analyzed by the Mann–Whitney *U*-test. Each symbol represents an individual sample, and horizontal lines indicate median values with error bars of the standard error. * *p* values ≤ 0.05 represented statistical significance.

In the absence of stimulation, innate and specific immune cells express all of the studied anti-inflammatory RBPs. *ZFP36* was an early expressed RBP. The RT-PCR analysis of PBMCs from LN patients and HC showed that the expression levels of *ZC3H12A* and *ZFP36* were similar (0.0703 ± 0.02 vs. 0.0721 ± 0.02 and 2.37 ± 0.73 vs. 2.41 ± 0.39) (Figure 1A,B). However, the mRNA expression levels of *RC3H1* were significantly lower in LN patients than in HC (0.0162 ± 0.005 vs. 0.0408 ± 0.01, *p* = 0.015), whereas the level of *RC3H2* was higher (without a statistical significance) in LN PBMCs than in the HC (0.239 ± 0.13 vs. 0.0434 ± 0.01, *p* = 0.627) (Figure 1C,D). We observed a positive correlation between the mRNA expression of *ZC3H12A* and *RC3H1* in the LN patients group (*p* = 0.001, r_s_ = 0.9).

**Figure 2:**
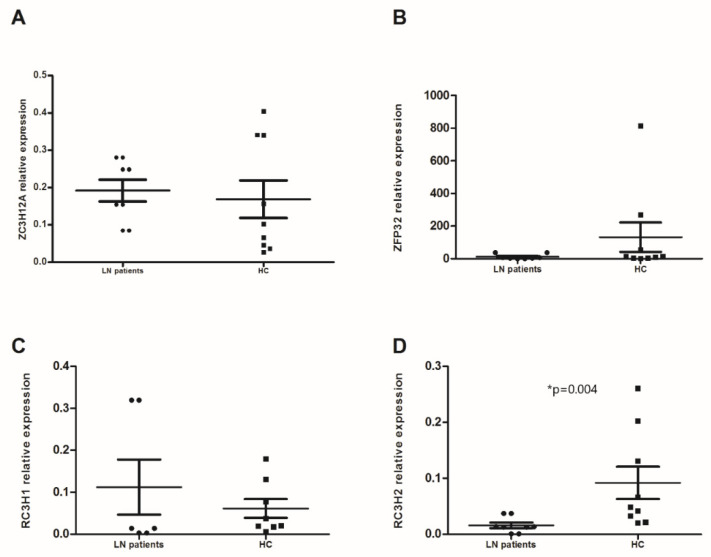
mRNA levels of anti-inflammatory RNA-binding proteins (RBPs) in polymorphonuclear neutrophils (PMNs): mRNA expression levels of (**A**) *ZC3H12A* (Regnase-1 gene), (**B**) *ZFP36* (TTP gene), (**C**) *RC3H1* (Roquin-1 gene), and (**D**) *RC3H2* (Roquin-2 gene) were determined by semi-quantitative RT-PCR in PMNs from lupus nephritis (LN) patients (n = 9) and healthy controls (HC) (n = 9) after normalization with GAPDH mRNA level. Data were analyzed by the Mann–Whitney *U*-test. Each symbol represents an individual sample, and horizontal lines indicate median values with error bars of the standard error. * *p* values ≤ 0.05 represented statistical significance.

Changes in expression of the 4 RBPs studied were also assessed in PMNs. Our results showed that only *RC3H2* was significantly higher in PMNs from LN patients compared to HC (0.091 ± 0.029 vs. 0.015 ± 0.007, *p* = 0.004) (Figure 2D). We noted a large gap in the *ZFP36* expression between the two groups but without a statistical significance (11.86 ± 8.52 vs. 132.23 ± 89.96, *p* = 0.123) (Figure 2B). The expression levels of *ZC3H12A* and *RC3H1* were similar (0.191 ± 0.044 vs. 0.168 ± 0.05 and 0.111 ± 0.1 vs. 0.061 ± 0.022) (Figure 2A,C). In the LN patients’ group, we observed a positive correlation between the mRNA expression of *ZFP36* and *RC3H2* (*p* = 0.017, r_s_ = 0.8).

The differential expression of RBPs studied between LN patients and HC in the subtype immune cells is summarized in Figure S2.

### 3.3. Target Genes Expression and Correlation with RBP

In basal conditions, the mRNA gene expression of *TNF-α*, *Il-6*, and *ICOS* revealed no statistically significant difference between PBMCs of LN patients and HC (0.155 ± 0.064 vs. 0.169 ± 0.041, 0.093 ± 0.07 vs. 0.048 ± 0.016, 0.02 ± 0.008 vs. 0.028 ± 0.004, respectively). The same results were shown in PMNs, excluding the *ICOS* gene (4.4 ± 4.28 vs. 0.148 ± 0.0063 and 0.0013 ± 0.0006 vs. 0.011 ± 0.00326, respectively). To investigate the general relationship between RBPs and their target genes at the transcriptional regulation level, we examined the correlation coefficient between the expression of RBPs and their targets in PBMCs and PMNs. Interestingly, in PBMCs, the most of the RBPs studied (*ZC3H12A*, *ZFP36,* and *RC3H1*) were only correlated with *TNF-α* (*p* = 0.001, r_s_ = 0.929; *p* = 0.002, r_s_ = 0.905; and *p* = 0.004, r_s_ = 0.881; respectively). This correlation remained in PMNs only with *RC3H1* (*p* = 0.003, r_s_ = 0.798).

### 3.4. Correlation between RBP Gene Expression in LN Patients and Their Clinical and Serological Features

In the two different cell types, no significant correlation was found between the expressed levels of the studied genes and clinical and serological features in LN patients, except with malar rash in PBMCs. Indeed, LN patients with concurrent skin rash expressed a significantly lower level of *ZC3H12A* compared to LN patients without malar rash (0.021 ± 0.001 vs. 0.139 ± 0.01, *p* = 0.02). The same observation was noted when focusing on the *IL-6* level (0.0026 ± 0.00027 vs. 0.0843 ± 0.021, *p* = 0.015). No association between the gene expression levels in LN patients and SLEDAI was found.

### 3.5. Global Change in the RBP Gene Expression Profile in Immune Cells Induced by LPS Stimulation

**Figure 3:**
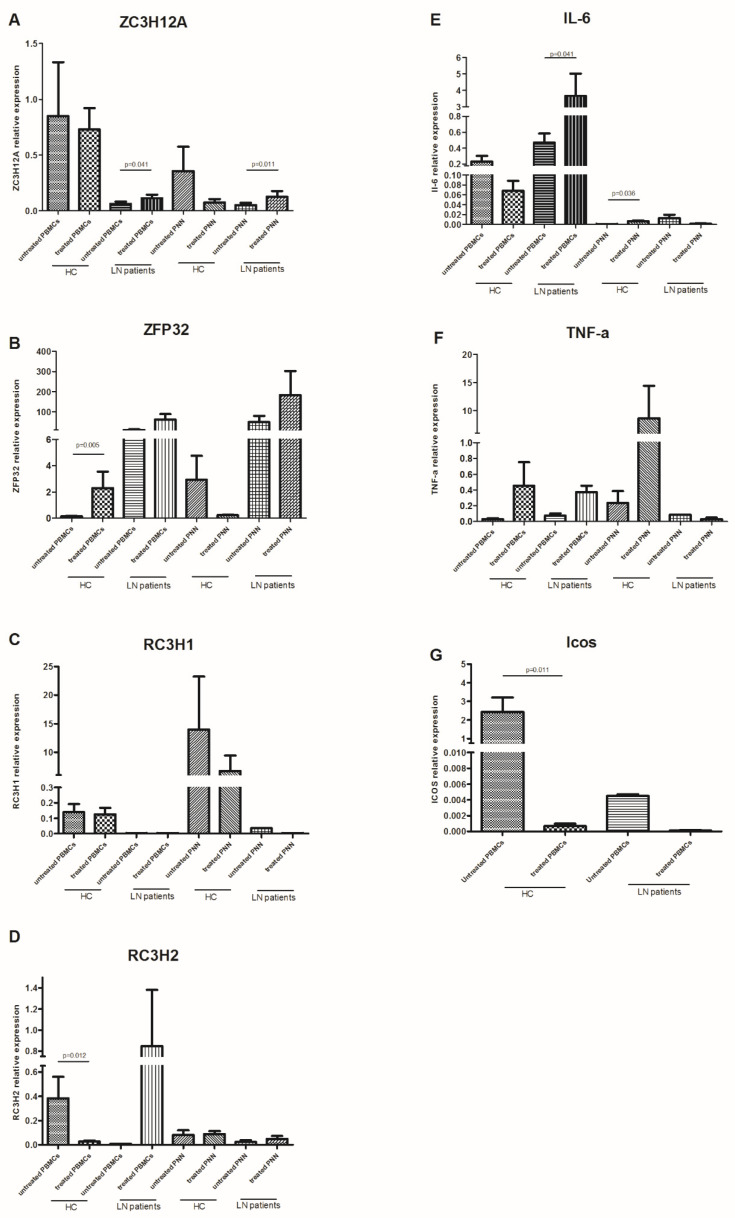
mRNA levels of anti-inflammatory RNA-binding proteins (RBPs) and their targets in peripheral blood mononuclear cells (PBMCs) and polymorphonuclear neutrophils (PMNs), with or without LPS stimulation: mRNA expression levels of (**A**) *ZC3H12A* (Regnase-1 gene), (**B**) *ZFP36* (TTP gene), (**C**) *RC3H1* (Roquin-1 gene), (**D**) *RC3H2* (Roquin-2 gene), (**E**) *IL-6*, (**F**) *TNF-α*, and (**G**) *ICOS* were determined by semi-quantitative RT-PCR in PBMCs and PMNs from lupus nephritis (LN) patients (n = 9) and healthy controls (HC) (n = 9), stimulated or not with LPS, after normalization with GAPDH mRNA level. Data were analyzed by the Mann–Whitney *U*-test. Each symbol represents an individual sample, and horizontal lines indicate median values with error bars of the standard error. *p* values ≤ 0.05 represented statistical significance.

To compare the effects of the LPS stimulation on immune cells, we investigated the expression of several RBPs (*ZFP36*, *RC3H1, RC3H2*, and *ZC3H12A*) and their respective targets (*IL-6*, *TNF-α,* and *ICOS*) in unstimulated and stimulated immune cells. Indeed, LPS-treated PBMCs expressed significantly higher levels of *ZC3H12A* and *RC3H1* compared to untreated PBMCs from LN patients (*p* = 0.041) (Figure 3A,D), though, the *IL-6* level was significantly higher (*p* = 0.041) (Figure 3E). However, PBMCs from HC expressed significantly higher levels of *ZFP36* and *RC3H2* (*p* = 0.005 and *p* = 0.012, respectively) (Figure 3B,D). This elevation concurred with a significant decrease in *ICOS* expression level (*p* = 0.011) (Figure 3G). No significant difference was revealed concerning *ZC3H12A* gene expression (Figure 3A). On the other hand, PMNs showed a different expression profile after LPS stimulation. *ZC3H12A* was strongly increased in treated LN patients’ PMNs compared to untreated ones (*p* = 0.011), while a barely significant decrease in *Il-6* was noted (*p* = 0.063) (Figure 3A,E). Conversely, this cytokine was significantly under-expressed in HC’ PMNs after LPS treatment (*p* = 0.036) (Figure 3E). 

The *RC3H1* and *TNF-α* gene expression did not differ after LPS stimulation either in PBMCs or PMN, between LN patients and HC.

### 3.6. RBP Expression in Renal Biopsies

**Figure 4:**
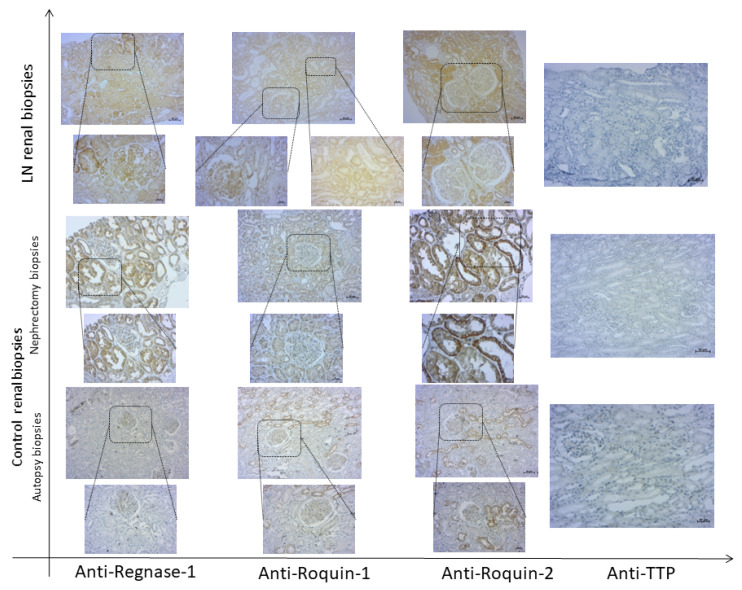
Expression of RNA-binding proteins (RBPs) in renal tissue of active lupus nephritis (LN) patients and controls. Representative immunohistochemical staining of Regnase-1, Roquin-1, Roquin-2, and TTP in a renal biopsy from an active LN patient and both nephrectomy and autopsy control renal tissues. Top row: 100× magnification; bottom row (insets): 200× magnification.

All renal biopsies, independently of histological diagnosis, showed variable degrees of tubular and glomerular positivity for Roquin-1, Roquin-2, and Regnase-1 markers, with a predominantly pronounced Regnase-1 expression (Figure 4).

**Figure 5:**
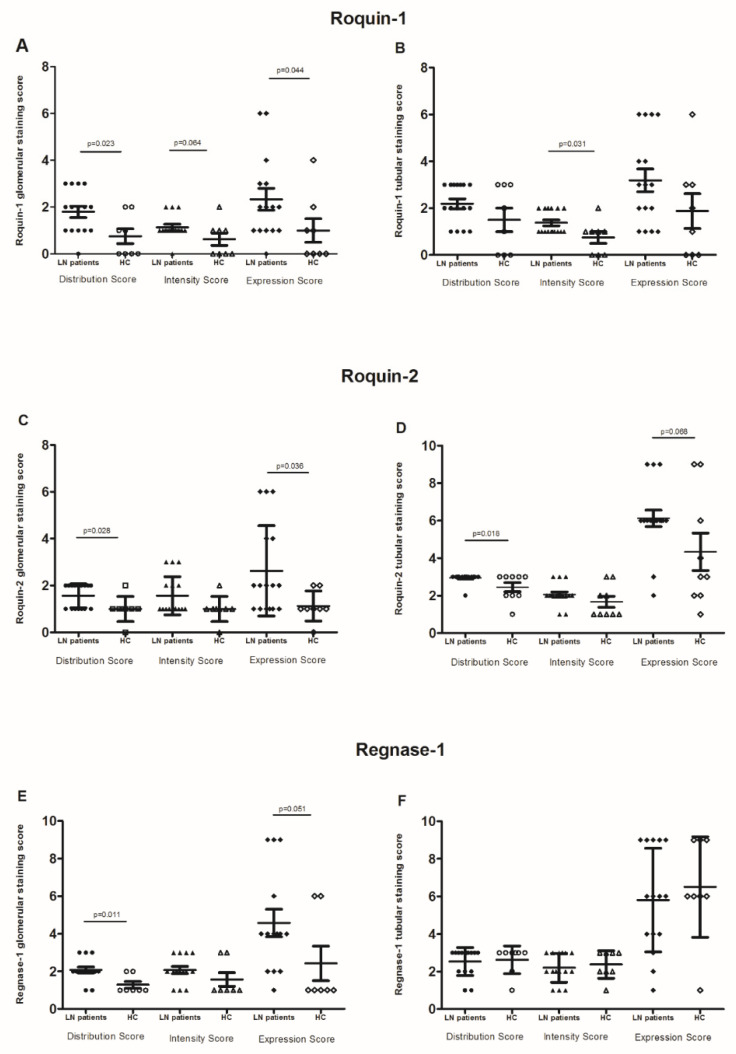
RNA-binding proteins (RBPs) expression: (**A**,**B**) glomerular and tubular Roquin-1 expression, (**C**,**D**) glomerular and tubular Roquin-2 expression, (**E**,**F**) glomerular and tubular Regnase-1 expression, in lupus nephritis (LN) patient biopsies compared to control biopsies. The mean expressions are represented with standard deviation (s.d.) LN renal biopsies (n = 18) and control renal biopsies (n = 9). Data were analyzed by the Mann–Whitney *U*-test. Each symbol represents an individual sample, and horizontal lines indicate median values with error bars of the standard error. *p* values ≤ 0.05 represented statistical significance.

IHC examination revealed that the LN patients’ renal biopsies displayed an intense and diffuse Roquin-1 and Roquin-2 staining in glomeruli, generating a higher expression score in LN patients compared to controls (2.33 ± 0.2 vs. 1 ± 0.5 and 2.6 ± 0.48 vs. 1.12 ± 0.22; *p* = 0.044 and *p* = 0.038, respectively) (Figure 5A,C). 

Furthermore, the glomerular distribution/intensity of Regnase-1 ranged from focal to diffuse, revealing that its expression was barely significantly higher in LN patients compared to controls (4.57 ± 0.72 vs. 2.4 ± 0.92, *p* = 0.051). (Figure 5E).

In tubules, Roquin-1 immunostaining intensity was significantly higher in LN biopsies than in control specimens (1.37 ± 0.12 vs. 0.75 ± 0.25, *p* = 0.001). However, the distribution score was similar between both groups of biopsies. Therefore, there was no difference in the Roquin-1 expression score (Figure 5B). Contrariwise, we revealed an increase in Roquin-2 expression score in LN patients compared to controls (6.11 ± 0.43 vs. 4.33 ± 1, *p* = 0.068) (Figure 5D). Regnase-1 tubular distribution, intensity, and the resulting score did not show any difference between LN patients and controls (Figure 5F).

When regarding RBPs expression labeling between tubules and glomeruli, the Spearman correlation test showed a positive correlation between glomerular distribution/intensity, and score expression of Roquin-1 and Regnase-1 (*p* = 0.004, r_s_ = 0.613; *p* = 0.038, r_s_ = 0.467 and *p* = 0.038, rs = 0.467, respectively). The same observation was found between Roquin-1 and Roquin-2 in tubular staining (*p* = 0.037, r_s_ = 0.428; *p* = 0.011, r_s_ = 0.512 and *p* = 0.004, r_s_ = 0.564, respectively). On the other hand, we did not reveal any correlation between RBPs expression in glomeruli and their correspondents in tubules, except Roquin-2, which showed a positive correlation between glomerular and tubular diffusion (*p* = 0.026, r_s_ = 0.453). 

Strangely, microscopic analysis of anti-TTP labeling on the same kidney sections showed very weak glomerular and tubular labeling in controls, especially in the smooth muscle cells of the walls of the blood vessels adjacent to the glomeruli, and the absence of its expression in LN patients’ renal biopsies (Figure 4).

**Figure 6:**
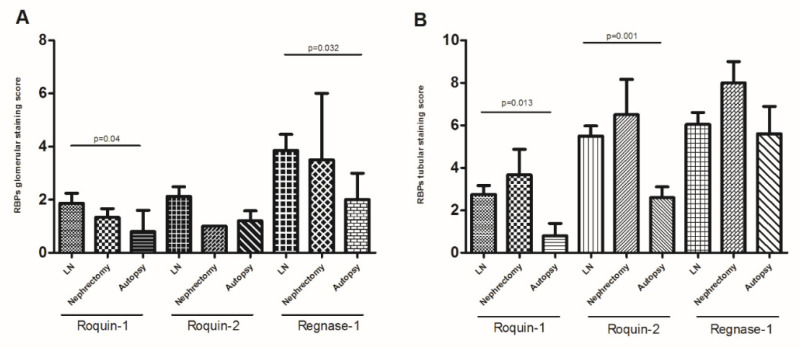
RNA-binding proteins (RBPs) expression: (**A**) glomerular Roquin-1, Roquin-2, and Regnase-1 expression, (**B**) tubular Roquin-1, Roquin-2, and Regnase-1 expression, in lupus nephritis (LN) patient biopsies compared to control nephrectomy and autopsy biopsies. The mean expressions are represented with standard deviation (s.d.) LN renal biopsies n = 18, controls nephrectomy biopsies n = 5, controls autopsy biopsies n = 4. Data were analyzed by the Mann–Whitney *U*-test. Each symbol represents an individual sample, and horizontal lines indicate median values with error bars of the standard error. *p* values ≤ 0.05 represented statistical significance.

When dividing the controls’ renal biopsies into 2 subgroups: autopsy and nephrectomy, we found persistence of the significant difference of glomerular Regnase-1 labeling in LN biopsies compared to autopsy biopsies (4.5 ± 0.72 vs. 2 ± 1, *p* = 0.032). Roquin-1 was significantly more expressed in the glomeruli and tubules of LN biopsies than in autopsy controls (2.33 ± 0.46 vs. 0.8 ± 0.8 and 3.18 ± 0.48 vs. 0.8 ± 0.58, *p* = 0.04 and *p* = 0.013, respectively), but Roquin-2’s significant expression difference was found solely in the tubules (6.11 ± 0.43 vs. 2.6 ± 0.5, *p* = 0.001) (Figure 6A). In contrast, a comparison of LN renal biopsies with those from nephrectomy sources revealed no significant differences for either protein expression at both glomerular and tubular levels (Figure 6B).

Regarding inter-subset analysis, the RBPs studied expression score was higher in non-proliferative group G2 compared to severe proliferative group G1 either in glomeruli and tubules, without a statistical significance.

### 3.7. Correlation between RBP Expression in LN Biopsies and Histological Features of These Biopsies

In our study, C1q along with IgG and C3 deposition was present in all LN biopsies. We investigated the correlation between the expression of different RBPs studied, except TTP, and the immunofluorescence and histological test results on these biopsies. Our results showed that tubular Roquin-2 staining was significantly higher in the presence of mesangial IgM deposits than in negative LN biopsies (7 ± 0.5 vs. 4.83 ± 0.74, *p* = 0.034). However, glomerular Roquin-2 expression was significantly higher in albumin-positive LN patients compared to negative ones (4 ± 0.81 vs. 1.75 ± 0.61, *p* = 0.029). 

Interestingly, the glomerular Roquin staining was shown to be inversely correlated with the LN activity index score. Indeed, the glomerular Roquin-1 expression correlated positively with higher activity index score (*p* = 0.002, r_s_ = 0.826), whereas the glomerular Roquin-2 expression was adversely correlated (*p* = 0.047, r_s_ = −0.582).

## 4. Discussion

In human and animal models, several signaling pathways are implicated in the pathogenesis of kidney disease such as inflammatory immune responses. Nonetheless, the understanding of the inflammatory and immunological pathways in LN illness, caused by renal impairment, lags considerably behind that of other SLE complications. 

To the best of our knowledge, this is the first human study to investigate the implication of different RBPs (Regnase-1, TTP, and Roquins) in the control of the immune-inflammatory responses in LN disease. We showed a significant increase in the expression of different studied RBPs either in immune cells, with or without a short LPS stimulation time, or in the glomeruli and tubules of renal biopsies of LN patients at different stages of the disease compared to controls. Roquins were the RBPs that showed the most significant difference, with reversed expressions between PBMCs and PMNs. Indeed, in comparison to controls, PBMCs showed lower amounts of *Roquin-1* gene level, but PMNs exhibited lower levels of *Roquin-2*, in LN patients. This observation did not corroborate the study of Vogel et al. showing that Roquin-2 is five times lower expressed than Roquin-1 in protein extracts from T CD4^+^ cells [24] and comfort overlapping functions of the two proteins [25]. It has been suggested that Roquin-2 could compensate for the action of Roquin-1 when it is absent and that redundant activity of Roquin-2 is inhibited in the presence of mutated Roquin-1 or WT in mouse models. The nature of this inhibition is unknown but could involve dominant-negative effects or the following competition for a shared and limiting factor [24]. On the other hand, our results confirm a correlation between Roquin-1 and TNF-α, and a regulating relation between Roquin-2 and ICOS after LPS stimulation, especially in HC. This observation agrees with different experiments demonstrating that these evolutionarily paralogous proteins work together to dampen critical mRNAs mediating inflammation and autoimmunity, including ICOS and TNF-α [26]. In kidney tissue, the inflammation site in LN, our results showed the presence of the Roquin-1 and Roquin-2 in both glomerular and tubular cells, with a significant increase in the latter. This observation is supported by studies published in the database (The Human Protein Atlas), but in contrast, Vogel et al. showed that the Roquins are weakly detectable in kidney extracts from wild-type mice [24]. Moreover, we found that Roquins expressions are higher in LN biopsies than in controls. Taking account of other murine studies that reported the loss of function of Roquins (caused by the point mutations) leads to the installation of glomerulonephritis with infiltration of autoreactive T and B cells and deposition of immune complexes in the kidneys [25,27,28]. We speculate that Roquins expression persists in LN kidneys to modulate the inflammation exhibited. This hypothesis is reinforced by the Roquins correlation with activity index and with the deposition of immune complexes, specifically for Roquin-1. Collectively, Roquins could play an important regulatory function in LN disease as a predictor of kidney damage. 

Other studies showed the development of severe autoimmune inflammatory diseases characterized by the infiltration of immune cells to various organs as well as the production of auto-Abs in Regnase-1-deficient mice [28]. This observation was not corroborated with our results which showed no difference in the expression of Regnase-1 in immune cells between LN patients and HC and its respective target, with a general increase in this expression in PMNs compared to PBMCs (*p* = 0.003). Moreover, Matsushita et al. showed, in mice macrophages, that Regnase-1 degrades a set of cytokine mRNAs (such as IL6) which are induced upon TLR stimulation [28]. Our human study showed that the level of this RBP mRNA was tightly controlled under TLR-4 signaling in both PBMCs and PMNs cells of LN patients and confirmed its correlation with TNF-α but not with IL6. This divergence may be explained by the difference between human and mouse metabolism. 

The revelation of the expression and localization of Regnase-1 in human kidney tissue sections, by IHC staining, showed tubular and more accentuated glomerular expressions in LN biopsies compared to controls. This glomerular expression was correlated with the activity index of the disease. A recently published study by Li et al. assessed the involvement of this RBP in acute glomerulonephritis (AGN) by murine in vivo experiments, which showed that Regnase-1 deficient mice present an exaggerated AGN with a more severe renal dysfunction and exaggerated tubular atrophy with an inflammatory cellular infiltration in the tubular cells compared to control mice [29].

Interestingly, when subdividing the control group into subgroups according to the origin of the renal biopsies, our IHC results showed a high expression of Regnase-1 and Roquins especially in non-malignant regions of the biopsies taken from nephrectomy specimens, unlike autopsy biopsies. This observation suggests the involvement of these RBPs in renal cancer pathology as supported by other studies, which revealed an important role of Regnase-1 in the regulation of angiogenesis and metastasis of renal cell carcinomas [30], and have involved the Roquins role in tumor suppression [31,32]. On the other hand, Pospiech et al. hypothesized that variation in *ZC3H12A* may be associated with the risk of clear cell renal cell carcinoma development [30]. To seek a genetic background of susceptibility for SLE, we evaluated the association of two potentially functional SNPs rs34796867 and rs34031609 of the *ZC3H12A* gene with the susceptibility to SLE in the Tunisian population by the candidate gene approach. Our results demonstrated no association between these polymorphisms and SLE disease (data not shown).

In addition to Regnase-1 and Roquin-1, PMNs expressed an increased level of TTP compared to PBMCs (*p* = 0.001), suggesting a differential expression according to the immune cell type. Our findings are supported by two other studies that demonstrated increases in the expression of these RBPs at the level of PMNs in mice and healthy donors [15,33]. Based on the capacity of PMNs to change their gene expression during inflammatory responses, compared to other immune cells, we suggest that these cells express the RBPs to control their apoptosis in chronic inflammatory conditions, given the involvement of these proteins in cell death. Transcriptome-wide studies in immune cells are needed to substantiate our hypothesis.

To our knowledge, our study is the first to assess the absence of TTP renal expression in LN biopsies in different stages of the disease compared with a control group; albeit, two studies have enrolled the kidneys of patients with diabetic nephropathy (DN) [34,35]. These studies showed that TTP is weakly expressed in podocytes, glomeruli, and tubules from control and DN renal biopsies. The strongly attenuated expression of TTP in the LN kidneys requires a lot of research to dig into and explain the cause of changes in the behavior of the protein. 

Our focus on the early events following LPS stimulation, a TLR-4 ligand, showed that this stimulation alone did not induce a high RBPs expression, especially in LN immune cells. We cannot rule out its obvious effect as an activator, especially since our previous study demonstrate the TLR-4 up-regulated expression in tubular epithelial and glomerular cells of LN patients. Moreover, our previous study showed that the renal TLR-4 expression is expressed differently between LN classes and correlates negatively with the sclerosis index [23]. Furthermore, Ostareck et al. showed that in LPS-induced macrophages, post-transcriptional RNA alterations may provide a new layer of control influencing RBP binding and therefore the destiny of their target mRNAs [36]. 

In humans, some diseases have been linked to defects in RBPs functions, especially cancer but not yet in SLE and its related complications [17,37,38,39]. The discordance between a high level of anti-inflammatory RBPs and the presence of chronic inflammation in LN patients makes us think about the failure of the mode of action of these RBPs and leads us to believe that these RBPs can be phosphorylated or inhibited by pro-inflammatory RBPs, in particular AT-rich interactive domain-containing protein 5A (Arid5a), following competition for the same protein binding site, thus facilitating the initiation and efficiency of inflammatory mediator production. Indeed, Arid5a has the role of protecting the mRNA of IL6 from the action of Regnase-1 to maintain increased levels of IL6 at the origin of inflammatory conditions [40].

## 5. Conclusions

Given RBPs’ critical roles in immune regulation, recognition of pro-inflammatory cytokine mRNA and costimulatory molecules, and degradation before translation into proteins, RBPs could be a promising therapeutic pathway but faced with the scarcity of human studies, their role in autoimmune disease remains a point of debate requiring further investigation. 

## Figures and Tables

**Table 1:** Demographic, serological, and clinical characteristics of the LN patients included in our study.

Parameters	LN Patients (n = 27)
Gender: female/male	23/4
Age of onset years (mean ± SD)	9.25 ± 4.501
Clinical manifestations	
Photosensitivity	33.3%
Malar rash	33.3%
Thrombopenia	33.3%
Lymphopenia	50%
Hemolytic anemia	33.3%
Serological manifestations	
Positive Anti-nucleosome	38.3%
Positive Anti-dsDNA	50%
Positive anti-histone	33.3%
Positive anti-SSA	50%
Positive anti-SM	50%
C3, g/L (median, min–max)	0.312 g/L (0.05–0.76)
C4, g/L (median, min–max) Histological details of LN patient’s Class 3 4 4 8 6 2	0.149 g/L (0.05–0.39) III. Focal proliferative IV. Diffuse proliferative V. Membranous VI+V III+V. II +V

LN, Lupus Nephritis; C3 and C4, complement component; CRP, C-reactive protein; anti-dsDNA, anti-double strand DNA.

## Data Availability

Data available on request from the authors.

## Supplementary Materials

The following supporting information can be downloaded at: https://www.mdpi.com/article/10.3390/life12101474/s1, Figure S1. Labeling of islets of Langerhans in a section of the pancreas with anti-TTP (A), anti-Regnase-1 (B), anti-Roquin-1 (C), and anti-Roquin-2 (D) antibodies. Figure S2. 3D box illustration of mRNA levels of anti-inflammatory RNA-binding proteins (RBPs) of (A) ZC3H12A (Regnase-1 gene), (B) ZFP36 (TTP gene), (C) RC3H1 (Roquin-1 gene), and (D) RC3H2 (Roquin-2 gene), in different blood cells, between lupus nephritis (LN) patients (n = 9) and healthy controls (HC) (n = 9) after normalization with GAPDH mRNA level. Table S1: Sequences of gene-specific primers used in quantitative real-time PCR analysis.
