# Supplementary material for: Differential Expression of Anti-Inflammatory RNA Binding Proteins in Lupus Nephritis
Source: Life (Basel). 2022 Sep 23;12(10):1474. doi: 10.3390/life12101474 (PMC9605213; doi:10.3390/life12101474)
Supplement: Supplementary file 1 [file life-12-01474-s001.zip › life-1837222-supplementary.pdf]

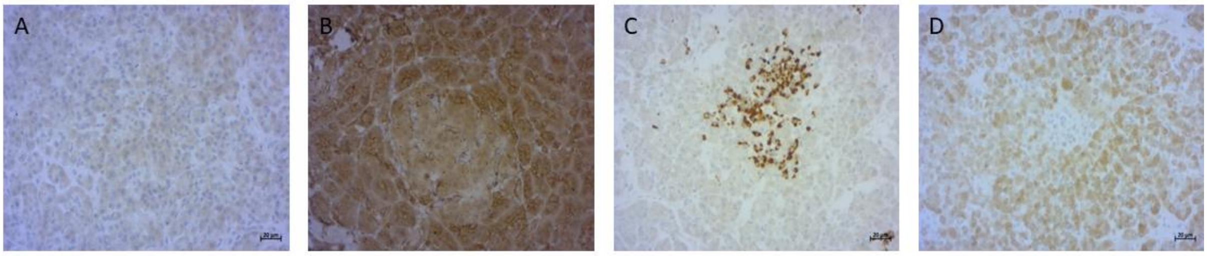

Figure S1. Labeling of islets of Langerhans in a section of the pancreas with anti-TTP (A), anti-Regnase-1 (B), anti-Roquin-1 (C), and anti-Roquin-2 (D) antibodies.

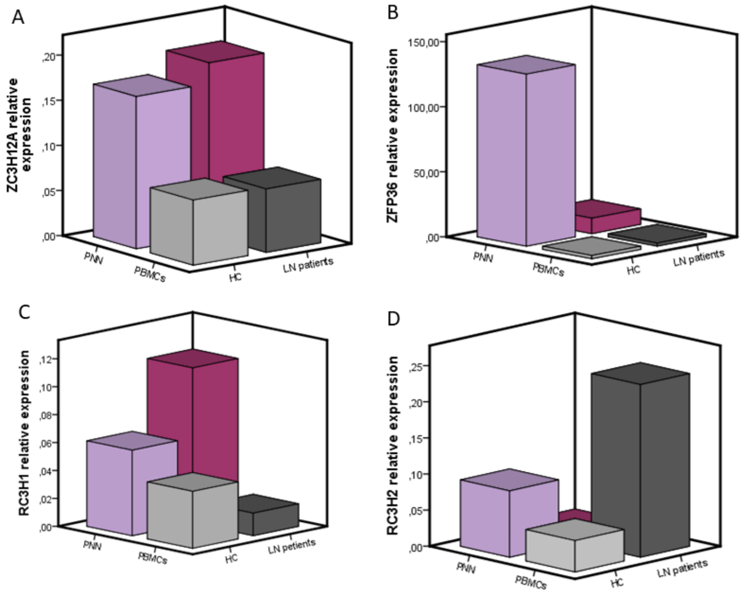

Figure S2. 3D box illustration of mRNA levels of anti-inflammatory RNA-binding proteins (RBPs) of (A) *ZC3H12A* (Regnase-1 gene), (B) *ZFP36* (TTP gene), (C) *RC3H1* (Roquin-1 gene), and (D) *RC3H2* (Roquin-2 gene), in different blood cells, between lupus nephritis (LN) patients (n=9) and healthy controls (HC) (n=9) after normalization with GAPDH mRNA level.

Table S1: Sequences of gene-specific primers used in quantitative real-time PCR analysis

Gene (NCBI Reference Sequence)	Gene-specific primers	Product Length
GAPDH (NM_001357943.2,NM_002046.7)	F: GCTCTCTGCTCCTCCTGTTC	123 pb
	R: CGCCCAATACGACCAAATCC	
ZC3H12A (NM_001323551.2, NM_001323550.2, NM_025079.3)	F: GCAGCGACCTGAGACCA	191 pb
	R: GTCTGTGATGGGCACGTC	
ZFP36 (NM_003407.5)	F: GGAGTGTCTTCCGAGGTTCTT	241 pb
	R: GCTACTTGCTTTTGGAGGGTAAT	
RC3H1 (NM_172071.4, NM_001300852.1, NM_001300851.1, NM_001300850.1)	F:GGCAGCTCGATCTTTAGGTG	381 pb
	R:TGTTCTCGCCGCAGAGCTTCA	
RC3H2 (NM_018835.5, NM_001354478.2, NM_001354482.2, NM_001354479.2, NM_001354486.2, NM_001100588.3)	F:GCGTTGAGGATTTGGCACTC	156 pb
	R:GCATGGCTCTTACACGACCT	
TNF-α (NM_000594.4)	F: CCAGGCAGTCAGATCATCTTCTC	150 pb
	R: AGCTGGTTATCTCTCAGCTCGAC	
IL-6 (NM_001371096.1, NM_001318095.2, NM_000600.5)	F: CAATCTGGATTCAATGAGGAGAC	118 pb
	R: CTCTGGCTTGTTCCTCACTACTC	
ICOS (NM_012092.4)	F: TTTGAACACTGAACGCGAGG	119 pb
	R: CAGAACCATTGATTTCTCCTGTT	

glyceraldehyde-3-phosphate dehydrogenase (GAPDH), zinc finger CCCH-type containing 12A (ZC3H12A), zinc finger protein 36 (ZFP36), ring finger and CCCH-type domains 1 (RC3H1), ring finger and CCCH-type domains 2 (RC3H2), tumor necrosis factor (TNF), interleukin 6 (IL-6), inducible T cell costimulator (ICOS). Forward (F), Reverse (R).
